# PCR-based versus conventional stool tests in children with diarrhea who underwent solid organ transplantation or hematopoietic stem cell transplantation

**DOI:** 10.1097/MD.0000000000035206

**Published:** 2023-09-22

**Authors:** Seewalee Sidafong, Pornthep Tanpowpong, Sophida Boonsathorn, Usanarat Anurathapan, Songkiat Chantarogh, Suporn Treepongkaruna

**Affiliations:** a Department of Pediatrics, Faculty of Medicine Ramathibodi Hospital, Mahidol University, Bangkok, Thailand.

**Keywords:** cytomegalovirus, diarrhea, enteric infection, immunosuppression, transplantation

## Abstract

Infectious diarrhea is a common problem among post-transplant recipients. Compared to conventional tests, polymerase chain reaction (PCR)-based stool tests have been shown to improve diagnostic yield but the aforementioned data in children remain limited. Our aims were to assess the detection rate of PCR-based tests in post-transplant children and compare with the conventional tests; and to investigate how these stool tests help in managing these children. We enrolled children aged 1 to 19 years who underwent solid organ transplantation or hematopoietic stem cell transplantation that remained on immunosuppressive agents and developed diarrhea ≥ 24 hours between January 2015 and February 2023. Besides stool tests, data on demographics, clinical characteristics and management were collected. We analyzed 68 patients and 92 episodes of diarrhea with PCR-based tests. PCR-based tests provided a detection rate of 41.8% versus 16.5% for the conventional tests. While conventional tests may detect a higher proportion of *Clostridiodes difficile* infection, PCR-based tests showed greater yields in detecting *Salmonella* spp. and viruses especially norovirus. PCR-based tests had an impact in management among 22/38 (58%) diarrheal episodes especially with *Campylobacter jejuni* and *C difficile*; and among 16 episodes that positive PCR-based tests had a minimal impact, the most common reason was due to the need for continuation of antimicrobial agents for concomitant site-specific infection (69%). Among transplanted children presenting with diarrhea, PCR-based tests provide a higher yield when compared with the conventional tests. The PCR-based stool tests may also further guide clinicians for providing proper antimicrobial agents.

## 1. Introduction

Diarrhea is a common problem among post-transplant recipients both in solid organ transplantation (SOT) and hematopoietic stem cell transplantation (HSCT). The prevalence ranges between 20% and 87% depends on the patient population and transplanted organs.^[1–7]^ In cases with infective diarrhea, transplanted recipients tend to be more severe than immunocompetent host because of their impaired immune function and underlying protein energy malnutrition in some cases.^[2]^ Therefore, an early and accurate diagnosis of enteric infection would likely provide a great opportunity for patients to obtain an effective and prompt management.^[6,8]^

When suspecting infective diarrhea in transplanted recipients, clinical symptoms and basic conventional stool tests such as microscopic examination for the ova and parasites, stool bacterial culture, or *Clostridiodes difficile* toxin have their own disadvantages. For example, microscopic examination for parasites severely suffers from low sensitivity and subjects to a significant operator-dependent issue.^[9]^ Moreover, conventional stool culture may not be able to detect the common pathogens causing community-acquired diarrhea such as *Campylobacter* spp. Therefore, highly sensitive tests are important not only to identify the infectious cause(s) but also offer an appropriate management and isolation to prevent further transmission especially in the inpatient setting. For example, norovirus is a common viral pathogen in HSCT recipients that has a high chance of transmission with a longer duration of diarrhea than most bacterial diarrhea and does not require the use of antibiotics.^[10]^

During the past decade, studies have shown a high rate of positivity of stool multiplex polymerase chain reaction-based gastrointestinal pathogen panel (GPP) compared with the conventional methods.^[8,11–17]^ The detection rate in children with stool GPP ranges 58% to 73% with a faster turnaround time.^[16–19]^ At our institution, Phrommas et al^[15]^ demonstrated that stool GPP detected pathogens in 34.2% but stool culture could detect organisms only in 5%. The study found that most episodes (68%) were “indefinite”; but enteric infection (either from bacteria or virus) was the most common known cause of diarrhea post SOT (15%), while the most commonly identified cause in HSCT recipients was gastrointestinal (GI) acute graft-versus-host disease (GVHD) (20%).

Most studies found that *C difficile*, enteropathogenic *Escherichia coli*, and norovirus were the 3 most detected organisms. While most organisms causing diarrhea can be detected by noninvasive stool tests, some pathogens such as cytomegalovirus (CMV) causing CMV GI disease would require mucosal biopsy and histopathology obtained from endoscopy as a gold standard. Recently, few studies reported that stool CMV polymerase chain reaction (PCR) provides acceptable sensitivity and accuracy when compared with the tissue biopsy^[20–22]^; however, the yield of stool CMV PCR remains debatable. Even PCR-based stool tests may provide higher sensitivity, the tests carry a high cost and may potentially demonstrate the non-viable part of the organisms which may not be pathogenic at the time of stool collection and can lead to an inappropriate management.

Studies comparing PCR-based stool tests with conventional tests among transplanted children residing in developing countries that may have different infection epidemiology compared with westernized countries remain limited. Therefore, our aims were to determine the detection rate of stool GPP and stool CMV PCR (i.e., PCR-based stool tests) in pediatric SOT and HSCT recipients, to demonstrate the rate of multiple organism detection, and to investigate how these tests help clinicians in managing these children.

## 2. Materials and methods

### 2.1. Study design and participants

The study was carried out into 2 parts (a retrospective part during January 2015 and July 2022 and a prospective part from August 2022 to February 2023). We included children aged 1 to 19 years who underwent SOT or HSCT and remained on immunosuppressive agents that developed a new onset of diarrhea for at least 24 hours prior to the enrollment. Patients who recently initiated common drugs causing diarrhea such as magnesium-, or phosphate-containing medications and supplementations, mycophenolate mofetil, or laxatives within the past 7 days, or patients who were admitted due to diarrhea within the past 14 days were excluded. The Institution Review Board of Faculty of Medicine Ramathibodi Hospital approved the study (COA. MURA 2021/1049). The caregiver/patient agreed and provided informed consent/assent to participate in the study.

Data on demographic and baseline characteristics including gender, age at enrollment and at transplantation, underlying disease, types of transplantation, and immunosuppressive drug at the onset of diarrhea were collected. We also collected clinical data and diagnostic evaluation from each episode of diarrhea, stool characteristics, associated symptoms as well as discontinuation or adding immunosuppressive drugs and antimicrobial agents during the diarrheal episode. Patient outcomes included diarrhea-related complications, length of hospital stay, and death were recorded.

### 2.2. Stool tests

For the prospective collection, each patient underwent conventional stool testing and PCR-based stool tests, including GPP and CMV PCR, at the same stool passage. The retrospective part of this study included all aforementioned available data of stool tests from the same diarrheal episode.

### 2.3. Conventional testing

The panel included 4 tests as the following: microscopic examination for ova and parasites; and bacterial cultures that required stool specimen at least 2 mL that was placed in the Cary-Blair transport medium within 24 hours. If not, the stool was kept in 2 to 8°C for <48 hours before placing on the medium. We used nutrient agar for *Salmonella typhi, Salmonella paratyphi* A, nontyphoidal salmonella, *Shigella* spp., diarrheagenic *E coli, Aeromonas* spp., *Plesiomonas* spp., *Vibrio* spp. Moreover, Alkaline Peptone Water was commonly used for the enrichment of *Vibrio cholerae* in the feces.; rapid chromatography immunoassay for detection of rotavirus, norovirus, and adenovirus; and *C difficile* glutamate dehydrogenase and toxin A and B using enzyme-linked fluorescent assay techniques.

### 2.4. Gastrointestinal pathogen panel

The xTAG GPP (Luminex Corporation, Toronto, Canada) used was a qualitative nucleic acid multiplex test that provides simultaneous detection and identification of multiple organism nucleic acids from 16 common gastroenteritis-causing agents as following: Adenovirus 40/41, astrovirus, *Campylobacter jejuni, C coli, C lari, C difficile* toxin A/B, *Cryptosporidium parvum, C hominis, Entamoeba histolytica, E coli* O157, enterotoxigenic *E coli* (ETEC) LT/ST, *Giardia lamblia*, norovirus GI/GII, rotavirus A, *Salmonella* spp., Shiga-like toxin producing *E coli* (STEC) stx 1/stx 2, *Shigella* spp. (*S boydii, S sonnei, S flexneri*, and *S dysenteriae*), *V cholerae,* and *Yersinia enterocolitica*. Stool GPP also required 2 mL of liquid stool and placed into screw top sterile container. Raw stool specimen was tested as soon as possible. If not processed immediately, the stool was also frozen at −70°C until further testing. The xTAG data analysis software usually provided a report within 5 to 24 hours.

### 2.5. Cytomegalovirus PCR

Stool CMV PCR was tested by using CMV real-time PCR by Abbott (Des Plaines, IL) with a limit of detection at 20 copies/mL.

### 2.6. Biostatistical analyses

Demographic data of post-transplant subjects are reported as a median with interquartile range, percentage, mean with standard deviation. Pearson Chi-square and Mann–Whitney *U* tests were used for categorical and continuous variables, respectively. The statistical significance was set at *P* value < .05.

## 3. Results

A total of 68 transplanted children (SOT 38% and HSCT 62%) were recruited with 50% females. The median age was 5 years (IQR 2, 11). We identified 92 diarrheal episodes, most had watery diarrhea. The most common symptoms accompanying diarrhea were fever, decrease appetite and abdominal pain. The median onset of diarrhea after transplantation was 115 (IQR 30, 409) days. The baseline data are shown in Table 1.

**Table 1:** Demographic data and clinical characteristics of the subjects.

Characteristics	N (%)
Overall patients (N = 68)	
Females	34 (50)
Transplanted organs	
LT	25 (36.7)
KT	1 (1.5)
HSCT	42 (61.8)
Age, years, median (IQR)	5 (2, 11)
LT	3 (1, 5)
KT	18
HSCT	8 (4, 14)
Characteristics of diarrheal episodes (N = 92)	
Number of detected episode(s) per patient	
1	68 (73.9)
2	10 (10.8)
3	4 (4.4)
>3	10 (10.8)
Stool characteristics	
Watery	67 (72.8)
Mucous	12 (13)
Mucous bloody	10 (10.9)
Bloody	3 (3.3)
Associate symptoms	83 (90.2)
Fever	62 (67.4)
Decrease appetite	37 (40.2)
Abdominal pain	29 (31.5)
Nausea/vomiting	24 (26.1)
Skin rash	14 (15.2)

HSCT = hematopoietic stem cell transplantation, KT = kidney transplantation, LT = liver transplantation.

We did not account 1 episode in a kidney transplantation recipient who had both negative PCR-based and conventional tests. Data of the 91 episodes are shown in Table 2. Pathogens were detected by the PCR-based stool tests in 38/91 episodes (41.8%) with 32 episodes being GPP-detected and 6 episodes had positive stool CMV PCR. The conventional test had 16.5% detection rate. When compared with the conventional tests, GPP had a statistically significant higher detection rate in the overall and among the retrospective episodes (*P* < .01). However, the detection rates of GPP and conventional tests became similar in the prospective period. Stool tests for diarrheal episodes in LT recipients had a higher detection rate when compared to the HSCT recipients. Diarrheal episodes in HSCT were noted to be earlier than the SOT recipients (median onset of 59 days [IQR 24, 167] vs 337 days [IQR 56, 1613], respectively, *P* < .001]). The median duration of overall diarrhea was 5 days. HSCT recipients were found to have longer duration of diarrhea when compared with the LT recipients (*P* < .001).

**Table 2:** Data on the diarrheal episodes (N = 91)*.

Tx	N	Episodes per patient	PCR-detected	GPP-detected	Conventional detected	*P* value GPP- vs conventional-detected	Duration, d, median (IQR)
Total	91	1.4	38 (41.8)	32 (35.2)	15 (16.5)	<.01	5 (3, 13)
LT	35	1.4	20 (57.1)	16 (45.7)	10 (28.6)	.03	4 (3, 4)
HSCT	56	1.3	18 (32.1)	16 (28.6)	5 (8.9)	<.01	8 (4, 17)
Retrospective (N = 67)							
Total	67	1.3	26 (38.8)	23 (34.3)	6 (9.0)	<.01	6 (4, 15)
LT	17	1.4	9 (52.9)	8 (47.1)	2 (11.8)	<.01	4 (4, 6)
HSCT	50	1.3	17 (34.0)	15 (30.0)	4 (8.0)	<.01	10 (4, 17)
Prospective (N = 24)							
Total	24	1.5	12 (50.0)	9 (37.5)	9 (37.5)	>.99	3 (3, 5)
LT	18	1.4	11 (61.1)	8 (44.4)	8 (44.4)	>.99	3 (3, 4)
HSCT	6	2	1 (16.7)	1 (16.7)	1 (16.7)	>.99	7 (6, 7)

GPP = gastrointestinal pathogen panel, HSCT = hematopoietic stem cell transplantation, LT = liver transplantation, PCR = polymerase chain reaction, Tx = type of transplantation.

* One kidney transplantation recipient without detectable organisms in any stool tests was not included in the table.

**Figure 1.**
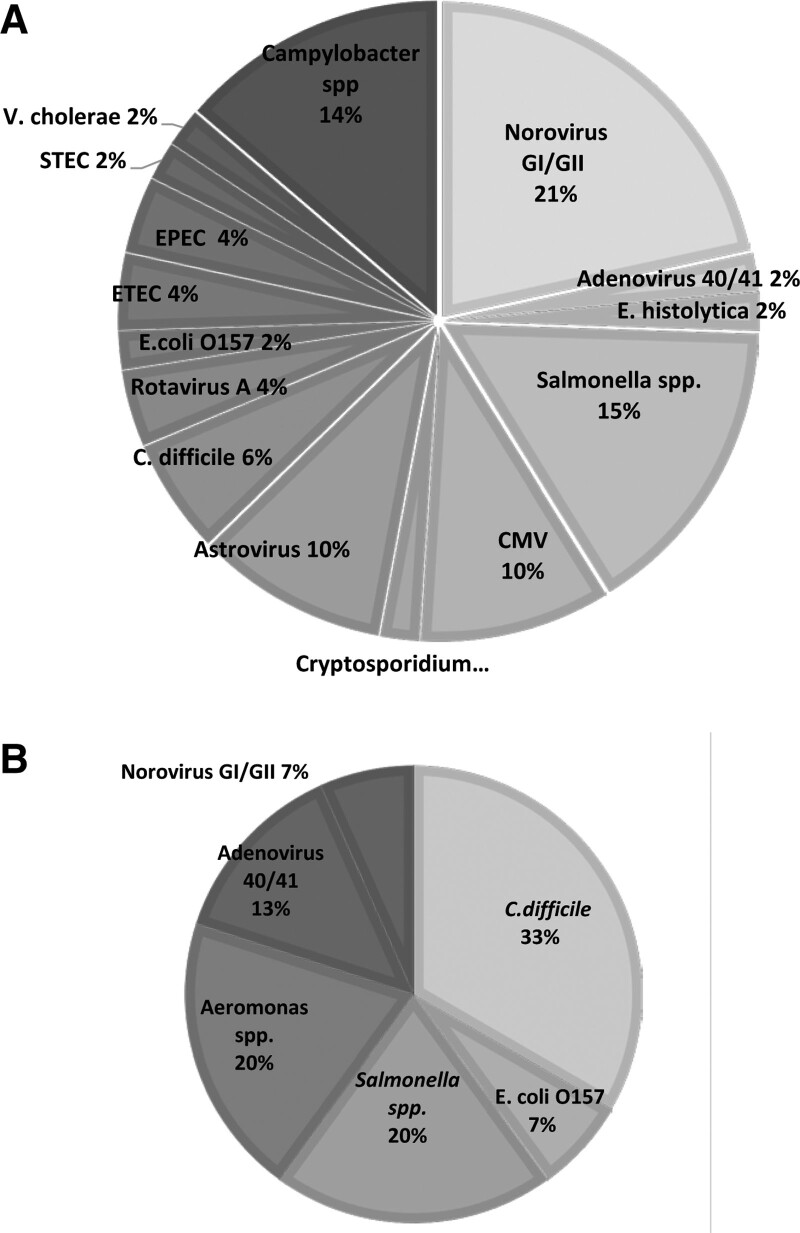
Pathogen detection in the PCR-based stool tests and conventional tests. PCR = polymerase chain reaction.

Among the PCR-detected episodes, 25 episodes (66%) had detected single organism and 13 episodes (34%) had detected multiple organisms. Among LT patients, the positive rates for detection of single and multiple organisms were similar but the HSCT patients had a higher rate for single organism detection (Table 3). We demonstrated that *Salmonella* spp., *Campylobacter* spp., and norovirus were the 3 most common co-occurring organisms (Table S1, Supplemental Digital Content, http://links.lww.com/MD/J766). Table 4 demonstrated detected pathogens in the study. The most common pathogens detected by GPP were norovirus (12.0%), *Salmonella* spp. (8.7%), *Campylobacter* spp. (7.6%) and astrovirus (5.4%). Of note, *Campylobacter* spp. and astrovirus cannot be detected by the conventional tests at our institution. While most common pathogens detected by the conventional tests were *C difficile* (9.8%), adenovirus (6.7%), and the same percentage of detection (3.3%) for *Salmonella* spp., *Aeromonas* spp., and norovirus. We found that various pathogens were only detected by the PCR-based stool tests such as *Campylobacte*r *spp., Cryptosporidium* spp. and astrovirus. Conversely, *Aeromonas* spp. could be detected only in the conventional stool culture. Figure 1 showed a more diverse detected pathogens in the PCR-based stool tests when compared with conventional tests in children who underwent SOT or HSCT.

**Table 3:** Rates of positive single vs multiple organisms detected by the PCR-based stool tests.

Type of organ transplantation	Positive single organism (%)	Positive multiple organism (%)	*P* value
LT (n = 35)	10 (28.6)	10 (28.6)	>.99
HSCT (n = 56)	15 (26.8)	3 (5.3)	<.001
Total (n = 91)	25 (27.2)	13 (14.1)	<.001

HSCT = hematopoietic stem cell transplantation, LT = liver transplantation, PCR = polymerase chain reaction.

**Table 4:** Various pathogens detected in study.

Pathogen	No. of pathogen (%)			
	Conventional tests		Stool GPP (n = 92)	
	All	Prospective (N = 25)	All	Prospective (N = 25)
Bacteria				
* Clostridioides difficile*	5/51 (9.8)	2 (8.0)	3 (3.6)	1 (4.0)
* Escherichia coli* O157	1/92 (1.1)	0	1 (1.1)	0
Enterotoxigenic *E coli*	N/A	N/A	2 (2.2)	1 (4.0)
Enteroaggregative *E coli*	N/A	N/A	0	0
Enteropathogenic *E coli*	N/A	N/A	2 (2.2)	2 (8.0)
* Salmonella* spp.	3/92 (3.3)	1 (4.0)	8 (8.7)	2 (8.0)
Shiga-like toxin producing *E coli*	N/A	N/A	1 (1.1)	0
* Shigella* spp.	0/92	0	0	0
* Vibrio cholerae*	0/92	0	1 (1.1)	0
* Yersinia enterocolitica*	N/A	N/A	0	0
* Campylobacter* spp.	N/A*	N/A*	7 (7.6)	1 (4.0)
* Aeromonas* spp.	3/92 (3.3)	3 (12.0)	N/A	N/A
Parasite				
* Cryptosporidium* spp.	N/A	N/A	1 (1.1)	1 (4.0)
* Entamoeba histolytica*	N/A	0	1 (1.1)	0
* Giardia lamblia*	N/A	0	0	0
Virus				
Adenovirus 40/41	2/30 (6.7)	2 (8.0)	2 (2.2)	1 (4.0)
Norovirus GI/GII	1/30 (3.3)	1 (4.0)	11 (12.0)	1 (4.0)
Rotavirus A	0	0	2 (2.2)	1 (4.0)
Astrovirus	N/A	N/A	5 (5.4)	5 (20.0)

GPP = gastrointestinal pathogen panel, N/A = not applicable.

* Selective culture media for *Campylobacter* spp. was not available at our institution.

Among 38 episodes with detected organisms in the PCR-based stool tests, we determined that PCR-based tests had a *significant* impact (i.e., initiation or cessation of antimicrobial agents) on the management in 22 episodes (58%) (details in Table 5) and had only *minimal* impact (i.e., PCR-based stool tests did not result in any changes in the management) in 16 episodes (42%) (details in Table 6). For episodes that positive PCR-based tests had a significant impact, 14/22 (64%) guided to a proper antimicrobial administration for various bacteria (e.g., *C jejuni, Salmonella* spp. enteropathogenic *E coli, C difficile*), 5/22 (22%) guided to a treatment for CMV, and 3/22 (14%) guided to a cessation of antimicrobial agents and/or supportive treatment. On the other hand, for episodes that positive PCR-based tests had a minimal impact on the management, the most common reason for not stopping the antimicrobial agents (11/16 episodes, 69%) was due to a concomitant site-specific infection during the same period of diarrhea (e.g., blood stream infection, intraabdominal collection, pneumonia, urinary tract infection or acute otitis media). We further compared the duration of diarrhea between the ones with versus without impact on the management of diarrhea and found no significant difference (mean [SD] of 6.7 [8.2] vs 11.4 [11.8] days, respectively, *P* = .20).

**Table 5:** Episodes that PCR-based tests had *significant* impact on the management (N = 22).

No.	Tx	Primary diagnosis	PCR-based tests	Conventional	Antimicrobial agents	Duration of diarrhea, d
1	LT	Acute diarrhea	*Campylobacter jejuni*	Neg	Azithromycin	2
			Norovirus			
2	LT	Crohn’s disease	*C jejuni*	Neg	Azithromycin	33
			Norovirus			
3	LT	Acute diarrhea	*C jejuni*	Neg	Azithromycin	4
			norovirus			
4	LT	Acute diarrhea	*C jejuni*	Neg	Azithromycin	3
			Norovirus			
5	LT	Acute diarrhea	*C jejuni*	*Aeromonas* spp.	Ciprofloxacin	4
6	HSCT	Acute diarrhea	*C jejuni*	Negative	Switch to azithromycin	3
7	HSCT	Acute diarrhea	*Salmonella* spp.	*Salmonella* group E	Add ceftriaxone	3
			Norovirus			
8	HSCT	Acute diarrhea	*Salmonella* spp.	Neg	Add ceftriaxone	5
			Adenovirus			
9	LT	Acute diarrhea	Enteropathogenic *Escherichia coli*	*Aeromonas* spp.	Ciprofloxacin	3
			Astrovirus			
10	LT	Acute diarrhea	Enteropathogenic *E coli*	*Aeromonas* spp.	Ciprofloxacin	4
			Astrovirus			
11	LT	Acute diarrhea	*Vibrio cholerae*	Neg	Switch to ciprofloxacin	4
12	LT	Acute diarrhea	*Clostridiodes difficile*	*C difficile* GDH: positive	Metronidazole	3
			CMV			
13	LT	*E coli* septicemia with acute diarrhea	*C difficile*	Neg	Previously on meropenem and add vancomycin	7
14	HSCT	Acute diarrhea	*C difficile*	Neg	Metronidazole	12
					Vancomycin	
15*	LT	CMV infection	CMV	*C difficile*	Ganciclovir	5
16*	HSCT	CMV GI disease with graft-versus-host disease	CMV	Neg	Ganciclovir	24
17*	HSCT	CMV reactivation with acute diarrhea	CMV	Neg	Ganciclovir	20
18*	LT	CMV GI disease	CMV	Neg	Ganciclovir	5
19*	LT	Acute diarrhea	CMV	Neg	Ganciclovir	4
20	LT	Acute diarrhea	Enterotoxigenic *E coli*	Neg	Supportive	2
21	LT	Acute diarrhea	Astrovirus	Neg	Supportive	2
22	HSCT	Acute diarrhea	Rotavirus	Neg	Off ceftriaxone (supportive)	4

CMV = cytomegalovirus, GI = gastrointestinal, HSCT = hematopoietic stem cell transplantation, LT = liver transplantation, PCR = polymerase chain reaction, Tx = type of transplantation.

* No.15-18 had endoscopy performed and reveal ulcers in the GI tract and pathology suggested of CMV GI disease, while no. 19 had positive stool CMV PCR but did not have endoscopy performed due to an early post-LT period.

**Table 6:** Episodes that PCR-based tests had *minimal* impact on the management (N = 16).

No.	Tx	Primary diagnosis	PCR-based tests	Conventional	Antimicrobial agents	Duration of diarrhea, d
1	LT	Acute diarrhea with septic shock	*Salmonella* spp.	*Salmonella*	Continue meropenem	5
			Astrovirus	group C		
			Norovirus			
2	LT	Abdominal collection	Salmonella spp.	Neg	Continue meropenem	4
			*Escherichia coli* O157			
			Enterotoxigenic			
			*E coli*			
3	HSCT	Septic shock with salmonella enteritis	S*almonella* spp.	*Salmonella*	Continue piperacillin-tazobactam	36
			*Entamoeba histolytica*	group B		
4	LT	Acute otitis media with diarrhea	*Salmonella* spp.	Adenovirus	Continue amoxicillin	2
			Adenovirus			
5	LT	Acute diarrhea	Rotavirus	Neg	Empirical ceftriaxone (3 d)	3
			*Cryptosporidium* spp.			
6	HSCT	Acute diarrhea	*Salmonella* spp.	Neg	Ceftazidime then ciprofloxacin (8 d)	2
7	LT	Bowel ischemia	*Salmonella* spp.	*Clostridiodes difficile* toxin A: positive	Prophylactic piperacillin-tazobactam	4
		GI bleeding				
8	HSCT	Acute diarrhea	*Campylobacter jejuni*	Neg	Ceftriaxone (3 d)	3
9	HSCT	Thrombotic microangio-pathy with GI bleeding	Shiga toxin-producing *E coli*	Neg	Continue meropenem	22
		Salmonella septicemia				
10	HSCT	Febrile neutropenia	Norovirus	Neg	Continue cefepime	4
11	HSCT	CMV reactivation	Norovirus	Neg	Continue foscarnet	6
12	HSCT	Pneumonia	Norovirus	Neg	Augmentin (7 d)	3
13	HSCT	UTI	Norovirus	Neg	Continue ceftazidime	17
14	HSCT	Pneumonia with septic shock	Norovirus	Neg	Continue meropenem	15
15	HSCT	Klebsiella UTI with septic shock	Norovirus	Neg	Continue meropenem	22
16	HSCT	Pneumonia	Astrovirus	Neg	Continue meropenem and amikacin	4

CMV = cytomegalovirus, GI = gastrointestinal, HSCT = hematopoietic stem cell transplantation, LT = liver transplantation, PCR = polymerase chain reaction, Tx = type of transplantation, UTI = urinary tract infection.

With regards to CMV, presumed CMV GI disease was noted in 8 episodes by confirmed positive histopathology of mucosal biopsies in 4 episodes, 3 episodes did not have a classic pathological feature of CMV GI disease, and 1 episode did not undergo endoscopy due to an early postoperative period after LT. All were initially treated with intravenous ganciclovir mainly due to CMV viremia (defined by plasma CMV PCR > 2000 copies/mL). Among 5 episodes with positive stool CMV PCR, one of them also had positive glutamate dehydrogenase for *C difficile* and received metronidazole (Patient No.12 in Table 5) with significant improvement of diarrhea and the responsible clinician decided to withhold endoscopy. Endoscopy was therefore performed in 4/5 and revealed ulcers in all 4 cases. However, classic histopathology was confirmed only in 1/4 of cases. Three cases with negative stool CMV PCR (but had CMV viremia) had histopathology consistent with CMV GI disease (Table S2, Supplemental Digital Content, http://links.lww.com/MD/J768).

## 4. Discussion

The purpose of this study was to compare the yield of PCR-based stool tests and conventional stool tests for detecting infectious etiologies in pediatric SOT or HSCT recipients with diarrhea. Data on transplanted children remains limited especially in the developing countries that may have different infection epidemiology and clinical practice. We found a positive rate of PCR-based stool tests of 41.8%, the rate that was lower than the previous PCR-based studies among transplanted recipients in developing countries.^[6,8,14]^ A pediatric study performed in the US found 69% detection rate among transplanted recipients using PCR-based test to detect 23 organisms,^[1]^ while our stool GPP test could detect only 16 organisms. The higher positive rate of conventional tests in the prospective part of the study (Table 2) was likely due to the ability to perform the tests at the same stool collection as the PCR-based stool tests.

Detection rates of the stool tests in LT recipients were higher than the HSCT recipients which would likely be due to that HSCT recipients may suffer from more diverse causes of diarrhea such as GVHD or pre-transplanted chemotherapy side effects. The characteristics between enteric infection and GVHD after HSCT in children are also indistinguishable. Therefore, the PCR-based stool test may demonstrate a higher rate of negative result among HSCT recipients as noted in our study. Furthermore, diarrheal episodes in the enrolled HSCT cases were also noted to be earlier than the SOT recipients which may also be due to an occurrence of acute GVHD that develops within the first 100 days after transplantation.^[23]^ We found a relatively similar rate of multi-organism positivity in the LT recipients (29% vs 32%) when compared to the US study.^[1]^ However, the HSCT recipients had a lower rate of multi-organism positivity (5% vs 20%) but the US study included only 5 HSCT cases with positive PCR-based stool test.

Regarding the detected pathogens, norovirus, *Salmonella* spp., *C jejuni.,* astrovirus, and CMV were commonly found in our study. A study by Pruksananonda et al^[24]^ performed in Bangkok, Thailand showed that among 1793 stool culture specimens sent, 10.8% revealed EPEC, and 2.9% had *C jejuni*; while of 1065 specimens tested for rotavirus antigen, 23.9% were positive. The pathogen profiles were quite different as compared to our study. Previous reports showed *C difficile*, rotavirus, and EPEC were the common pathogens,^[1,6,8]^ but our study detected those organisms only in 3.6%, 2.2%, and 2.2%, respectively. *C jejuni, Salmonella* spp., and norovirus were also the most common co-occurring organisms, similar to the studies from other centers.^[1,6,10]^ *C difficile* was one of the commonest co-infections in 2 large studies which might be due to the prolonged courses of antibiotics and hospital stay.^[1,10]^ Asymptomatic *C difficile* colonization was more commonly reported in young children aged < 2 years old when compared to older children. The positive results may therefore not be the true cause of diarrhea in infants and young toddlers.^[25]^ However, cases No. 12-14 in Table 5 were patients aged > 3 years which would not likely be subject to the issue of asymptomatic colonization as mentioned.

*Campylobacte*r spp., various *E coli* pathotypes, *Cryptosporidium* spp., and astrovirus would likely and only be detected by the stool GPP but not by most conventional stool tests. Some of the above organisms may require specific antimicrobial agents to alleviate the diarrheal symptoms. Conversely, most enteric viruses may just need supportive treatment without specific management. Stool GPP may therefore provide a strong benefit in detecting the aforementioned organisms and further guiding to an appropriate management in these children. The positive rate of *Aeromonas* spp. by conventional stool culture was approximately 3% which was similar to the recent studies.^[15,17,19]^ Aeromonas is one of the common pathogens that would require antibiotic treatment to shorten the symptoms, and it can only be detected via stool culture but not the PCR-based test at our institution.

Among the 38 episodes with detected organisms in the PCR-based stool tests, we determined that PCR-based tests had a significant impact on the management in 22/38 (58%), especially impacts on prescribing proper antimicrobial agents (19/22, 86%). However, the main outcome (i.e., duration of diarrhea) between the ones with versus without impact on the management showed no significant difference. Similar to the result from our study (Fig. 1), the PCR-based tests have been shown to detect higher variety of pathogens more than the conventional tests in previous studies performed in immunocompetent children and transplanted adult recipients.^[6,10]^

With regards to presumed CMV GI disease, among 5 episodes with positive stool CMV PCR, classic histopathology confirmed only in 1 of the 4 cases that underwent endoscopy, while the other 3 cases with negative stool CMV PCR had histopathology consistent with CMV GI disease. As all patients with presumed CMV GI disease had CMV viremia, these patients initially received ganciclovir before the endoscopy could be performed. Therefore, the “negative” classic histopathology may likely be because of the antiviral effect of ganciclovir. Stool CMV PCR may be considered as a noninvasive diagnostic tool for the diagnosis of CMV GI disease, but we cannot conclude that stool CMV PCR would replace gold-standard histopathology.^[20,22,26]^ A previous study by Prachasitthisak et al^[20]^ reveal that 3 of the 8 episodes of positive stool CMV PCR did not have a documented CMV GI disease defined by the histopathology.

This study carries some limitations. First, we are aware of the lack of conventional tests for some retrospective cases to compare with the PCR-based stool tests. Second, the study was carried out in a single center with relatively small patient population. Third, at the time of this study, our center did not have selective media used for *C jejuni* or rapid antigen tests for *Giardia spp.* and *Cryptosporidium* spp. Moreover, cost-effectiveness analyses for the tests, treatment, and outcome were not combinedly considered. With regards to the management of diarrhea, the decision was based on the primary responding physician, not the research investigators. Each physician may practice and manage infective diarrhea differently even noticing the same organism(s), but on the other hand, this situation would likely reflect the real-world practice. We therefore hope that data from this study add more useful data on the yield of PCR-based stool tests in transplanted children with similar patient settings.

## 5. Conclusions

PCR-based stool tests demonstrate a higher detection rate with greater variety of organisms when compared with conventional tests among pediatric transplanted recipients. The PCR-based test may also help guiding clinicians for further investigations and proper management, e.g., the use of antimicrobial agents. However, the impact on clinical outcomes such as duration of diarrhea and cost-effective analyses would merit further investigations through more follow-up studies.

## Author contributions

**Conceptualization:** Seewalee Sidafong, Pornthep Tanpowpong, Sophida Boonsathorn, Usanarat Anurathapan, Songkiat Chantarogh, Suporn Treepongkaruna.

**Formal analysis:** Seewalee Sidafong.

**Investigation:** Seewalee Sidafong, Pornthep Tanpowpong.

**Methodology:** Pornthep Tanpowpong, Sophida Boonsathorn, Songkiat Chantarogh.

**Supervision:** Pornthep Tanpowpong, Usanarat Anurathapan, Suporn Treepongkaruna.

**Validation:** Pornthep Tanpowpong.

**Writing – original draft:** Seewalee Sidafong.

**Writing – review & editing:** Pornthep Tanpowpong.

## Supplementary Material




