# Supplementary material for: PCR-based versus conventional stool tests in children with diarrhea who underwent solid organ transplantation or hematopoietic stem cell transplantation
Source: Medicine (Baltimore). 2023 Sep 22;102(38):e35206. doi: 10.1097/MD.0000000000035206 (PMC10519553; doi:10.1097/MD.0000000000035206)
Supplement: Supplementary file 1 [file medi-102-e35206-s001.docx]

**Supplementary Table 1** Data on types of organism based on the number of detected organisms (single organism vs. multiple organisms) from the PCR-based stool tests (N=38)

| Organism | Single organism  N (%) | Multiple organisms  N (%) |
| --- | --- | --- |
| Bacteria |  |  |
| *Clostridioides difficile* | 1 (4.4) | 2 (6.9) |
| *Escherichia coli* O157 | 0 | 1 (3.5) |
| Enterotoxigenic *Escherichia coli* | 1 (4.4) | 1 (3.5) |
| Enteropathogenic *E. coli* | 0 | 2 (6.9) |
| *Salmonella* spp. | 2 (8.7) | 6 (20.7) |
| Shiga-like toxin producing *E. coli* | 1 (4.4) | 0 |
| *Vibrio cholerae* | 1 (4.4) | 0 |
| *Campylobacter* spp*.* | 3 (13.4) | 4 (13.8) |
| Parasite |  |  |
| *Cryptosporidium* spp. | 0 | 1 (3.5) |
| *Entamoeba histolytica* | 0 | 1 (3.5) |
| Virus |  |  |
| Adenovirus 40/41 | 0 | 1 (3.5) |
| Norovirus GI/GII | 6 (26.1) | 5 (17.2) |
| Rotavirus A | 1 (4.4) | 1 (3.5) |
| Astrovirus | 2 (8.7) | 2 (6.9) |
| Cytomegalovirus | 5 (21.7) | 1 (3.5) |
