# Supplementary material for: PCR-based versus conventional stool tests in children with diarrhea who underwent solid organ transplantation or hematopoietic stem cell transplantation
Source: Medicine (Baltimore). 2023 Sep 22;102(38):e35206. doi: 10.1097/MD.0000000000035206 (PMC10519553; doi:10.1097/MD.0000000000035206)
Supplement: Supplementary file 2 [file medi-102-e35206-s002.docx]

**Supplementary Table 2** Data of 8 children with presumed CMV gastrointestinal disease

| No. | Tx | Stool CMV  PCR | Plasma CMV PCR (copies/mL) | Endoscopic  Findings | Pathology | Antiviral agent | Duration of diarrhea, d |
| --- | --- | --- | --- | --- | --- | --- | --- |
| 1 | LT | Pos | 9,240 | Two clean-based ulcers at duodenal bulb, multiple aphthous ulcers along colon | Mild chronic gastritis, Moderate active colitis at sigmoid colon | Ganciclovir | 5 |
| 2 | HSCT | Pos | 35,600 | Ulcers at rectosigmoid | CMV proctocolitis | Ganciclovir | 20 |
| 3 | LT | Pos | 42,500 | Ulcers at rectosigmoid | Unremarkable | Ganciclovir | 5 |
| 4 | HSCT | Pos | 4,720 | Multiple small ulcers at left colon,  rectosigmoid | Apoptosis of crypts at cecum  and colon | Ganciclovir | 24 |
| 5* | LT | Pos | 2,570 | N/A | - | Ganciclovir | 4 |
| 6 | HSCT | Neg | 3,500 | Erythematous mucosa at stomach and terminal ileum | CMV gastritis and some cytomegalic changes in the colonic mucosa | Ganciclovir | 8 |
| 7 | HSCT | Neg | 2,090 | Swollen mucosa at colon and rectum | CMV colitis at rectosigmoid | Ganciclovir | 34 |
| 8 | HSCT | Neg | 27,800 | Ulcers at the terminal ileum and colon | Cytomegalic changes with intranuclear inclusion and cell apoptosis | Ganciclovir | 13 |

CMV, cytomegalovirus; HSCT: hematopoietic stem cell transplantation; LT: liver transplantation; PCR, polymerase chain reaction, Tx; type of transplantation.

*This patient was presumed to have CMV GI disease from symptoms (i.e., diarrhea) and positive stool CMV PCR test but did not undergo endoscopy because the diarrheal episode occurred early in the postoperative period of liver transplantation.
